# Clinical Use of Infrared Thermography: Where Are We and Where Are We Going

**DOI:** 10.3390/medicina62061204

**Published:** 2026-06-22

**Authors:** Agnieszka Wnuk-Scardaccione, Jan Bilski

**Affiliations:** Department of Biomechanics and Kinesiology, Institute of Physiotherapy, Faculty of Health Sciences, Jagiellonian University Medical College, 8 Skawińska Street, 31-066 Krakow, Poland; agnieszka90.wnuk@uj.edu.pl

**Keywords:** medical thermography, non-invasive diagnostic, thermal camera, artificial intelligence, clinical validation

## Abstract

Medical infrared thermography, which involves the use of infrared thermal cameras for the non-invasive assessment of skin surface temperature distribution, has gained increasing interest in recent years as a tool supporting diagnosis and treatment monitoring. The aim of this article is to present the historical background and critically reassess the current role of infrared thermography in medicine, with particular emphasis on standardization as a key determinant of its clinical utility. This Perspective highlights the fundamental impact of methodological variability on diagnostic performance and reproducibility. A structured framework for standardization is proposed, encompassing patient preparation, environmental conditions, device parameters and calibration, image acquisition protocols, region-of-interest definition and analysis, as well as reporting and clinical interpretation. The analysis demonstrates how inconsistencies at each of these levels reduce measurement reliability, limit inter-study comparability, and weaken clinical confidence in infrared thermography. The article also addresses the growing availability of mobile thermal imaging systems and their integration with artificial intelligence, while emphasizing the need for stronger evidence-based support across all methodological domains. The presented analysis suggests that, despite existing limitations, medical infrared thermography holds considerable potential as a supportive clinical tool. However, its broader clinical implementation remains limited by several factors, with the lack of standardized protocols constituting a major and practically addressable translational barrier. Wider adoption will require standardization efforts alongside rigorous validation studies and application-specific interpretative guidelines. Addressing these challenges through technological advances and coordinated international standardization may facilitate meaningful progress over the next decade.

## 1. Introduction

Elevated body temperature has long been recognized as one of the most important clinical signs, although temperature assessment remained subjective until the 16th century. The origins of thermography (Infrared Thermography—IRT) date back to the work of Herschel, who discovered the thermal aspect of solar radiation. IRT was first applied in medicine in 1959 to study areas of increased heat associated with inflammation [1]. Initially, the examination took several minutes, and interpretation was complicated, with a lack of standardization. Subsequently, improvements in imaging conditions gradually produced higher quality thermograms, which are now a valuable complement to other imaging modalities such as ultrasound, MRI, and CT [2]. Essential progress in thermal imaging was made in the 1960s with the adaptation of military infrared thermal cameras for medical diagnostic applications. Despite this early promise, medical IRT soon experienced a decline in clinical use due to issues with detector sensitivity and competition from more established imaging methods [3]. Limited understanding of measurement conditions and the complex heat flows in humans often led to inaccurate results, causing disappointment among medical staff, particularly with false interventions in breast cancer treatment [4]. Interest in the technique began to recover with the introduction of modern cameras, increasing efforts toward methodological standardization, and the integration of artificial intelligence and machine learning for pattern recognition. At the same time, the growing demand for telemedicine and remote patient monitoring has renewed interest in the clinical potential of infrared thermography as a valuable complementary tool in modern healthcare. However, despite its increasing adoption, the broader clinical implementation of IRT remains limited by the lack of unified international standards regarding imaging protocols, data interpretation, and device calibration [5]. The aim of this Perspective is to critically evaluate the current state of standardization in the context of the historical use of IRT and to propose future directions for the development of this assessment method in order to enhance its clinical applicability. While several factors continue to limit the clinical translation of IRT, the lack of standardized protocols represents one of the most tangible and actionable barriers to its broader implementation.

## 2. Scope and Approach of This Perspective

This Perspective provides a conceptual and forward-looking overview of the use of infrared thermography in medicine. The presented narrative synthesis is based on a purposive and non-systematic selection of publications, including both seminal studies that established the foundations of the field and recent representative contributions reflecting current technological and clinical developments. The selected literature was chosen to illustrate three main domains: (i) the physical and biological principles underlying infrared thermography, (ii) methodological limitations and sources of variability affecting reproducibility and standardization, and (iii) the increasing role of artificial intelligence in IRT and possible future directions.

## 3. Physical and Biological Basis of Medical Infrared Thermography

IRT is based on the detection of infrared radiation emitted by the human body, primarily within the 8–14 μm wavelength range. As all objects above absolute zero emit infrared radiation, the homeothermic human body continuously generates measurable thermal emissions that can be captured by specialized detectors and converted into visible, quantifiable thermal images [6]. Infrared radiation emitted from the skin surface reflects underlying physiological processes, particularly blood flow and tissue perfusion [7]. Heat generated in deeper tissues is transported to the skin mainly through blood circulation; therefore, regional skin temperature largely depends on microcirculatory dynamics and vascular tone. Increased perfusion, such as during inflammation or vasodilation, raises local temperature, whereas reduced blood flow may lead to relative cooling [8]. Local metabolic activity also affects thermal emission by influencing tissue heat production. Increased metabolism and perfusion, such as during inflammation, may cause localized hyperthermia, whereas reduced metabolic activity or impaired blood supply can appear as cooler regions. Therefore, IRT provides indirect information about the interaction between perfusion, metabolism, and autonomic regulation rather than directly measuring these processes [9]. IRT has also been used to assess the activity of adipose tissue, particularly brown adipose tissue (BAT). BAT produces heat via non-shivering thermogenesis mediated by mitochondrial uncoupling proteins, and its activation can lead to subtle, localized increases in skin temperature detectable by IRT [10]. IRT is also associated with autonomic nervous system (ANS) activity and metabolic regulation. Cutaneous blood vessels are mainly controlled by sympathetic vasomotor fibers, which regulate vascular tone in response to thermoregulatory and stress stimuli. Sympathetic activation typically causes vasoconstriction and skin cooling, while reduced tone leads to vasodilation and warming. However, thermographic patterns reflect the combined influence of neural regulation, perfusion, tissue properties, and environmental factors rather than isolated ANS activity [11].

## 4. Standardization of Infrared Thermography in Clinical Practice

To properly interpret IRT in clinical settings, it is essential to recognize the multiple sources of variability that influence measurement accuracy and reliability. Environmental conditions such as ambient temperature, humidity, airflow, and external radiant heat can significantly alter skin surface temperature and confound results, making strict environmental control particularly important when clinically relevant differences are small [12]. In addition, patient-related factors such as skin characteristics, subcutaneous fat, hydration, and vascularization may affect thermographic measurements by altering the intensity and distribution of thermal signals [13]. In practice, these physiological sources of variability often relate to differences in age, body composition, and skin phenotype, which should be carefully considered during thermographic interpretation. In the following sections, we will discuss the key stages of the infrared thermography workflow—patient preparation, environmental conditions, device technical parameters and calibration, image acquisition protocols, region of interest (ROI) definition and image analysis, as well as reporting and clinical interpretation—in the context of how the current lack of global standardization at each of these levels affects measurement repeatability, limits inter-center comparability, and ultimately reduces clinical confidence in thermographic findings. In many clinical settings, reported inter-session variability approaches the magnitude of diagnostically relevant thermal asymmetries.

Patient preparation represents one of the most critical and, at the same time, most heterogeneous stages in IRT protocols. Despite its direct impact on measurement outcomes, there is currently no universally accepted standard defining pre-imaging conditions across clinical studies or institutions. As a result, substantial variability exists in how patients are instructed and prepared before data acquisition [14]. Differences such as: required acclimatization time, restrictions on physical activity, caffeine or nicotine intake, use of topical products, or recent exposure to temperature extremes can significantly influence cutaneous perfusion and, consequently, skin temperature distribution [15]. Even short-term physiological changes related to sympathetic activation or vascular reactivity may introduce measurable thermal variability that is unrelated to the underlying pathology. It also limits comparability between studies conducted in different centers, where patient preparation protocols are often inconsistent or insufficiently reported [16]. In musculoskeletal imaging, where IRT is frequently used to assess inflammatory processes, sports injuries, or chronic pain syndromes, insufficient patient standardization may lead to false-positive or false-negative thermal asymmetries. This is particularly problematic because diagnostic interpretation often relies on small bilateral temperature differences, making these protocols highly sensitive to external and physiological confounders [17].

Environmental conditions represent another critical source of variability in IRT and are among the most influential factors affecting measurement reliability. Despite their well-documented impact, environmental conditions in clinical IRT are still not governed by harmonized protocols, contributing to substantial inter-study and inter-center heterogeneity. Parameters such as ambient temperature, humidity, airflow, and external radiant heat sources directly influence cutaneous heat exchange and can alter skin surface temperature independently of underlying physiological processes. Even minor fluctuations in room conditions may introduce measurable changes in thermal distribution, particularly when diagnostic interpretation relies on small temperature differences (<0.5 °C) [18]. Lack of strict environmental standardization reduces reproducibility and may alter apparent thermal patterns even in otherwise comparable subjects. It also complicates comparison between studies, where environmental conditions are often inconsistently reported or controlled. Consequently, environmental variability remains a major obstacle to reproducibility and cross-study comparability [19].

In medical IRT, proper calibration of the imaging camera and appropriate technical parameters are essential to ensure reliable and clinically useful results [20]. Thermal cameras used in medicine therefore require regular radiometric calibration, most commonly performed using a black-body reference source with a precisely controlled temperature. This procedure enables correction of sensor drift and improves measurement accuracy and repeatability. Several technical parameters strongly influence diagnostic quality. High thermal sensitivity, expressed as the Noise Equivalent Temperature Difference (NETD), is necessary to detect subtle temperature differences associated with inflammation, vascular disorders, or tumor activity. In medical applications, the NETD should preferably be below 50 mK (0.05 °C), while high-performance systems may achieve values of 20–30 mK, enabling the detection of very small thermal asymmetries [21].

Medical thermal imaging systems commonly use detector resolutions of at least 320 × 240 pixels, whereas higher-end cameras with resolutions of 640 × 480 pixels or greater provide improved image detail and more precise localization of pathological changes. Detector stability, correct emissivity settings for human skin (approximately 0.98), and an appropriate spectral range, usually within 8–14 µm, are also crucial for obtaining reliable and repeatable measurements. Portable smartphone-based thermal cameras, such as compact devices attached directly to mobile phones, have become increasingly popular due to their low cost and accessibility. However, many of these systems offer lower spatial resolution, higher NETD values, and reduced radiometric stability compared with professional medical-grade thermal cameras [22].

Standardization of the image acquisition protocol is essential to ensure reliable, reproducible, and clinically comparable results. The protocol should include controlled environmental conditions, such as stable room temperature (typically 20–24 °C), low air circulation, and the absence of direct external heat sources. In addition, the protocol should define camera-related parameters, including imaging distance, viewing angle, emissivity settings, calibration status, and detector stabilization time. Standardized patient positioning and image acquisition procedures are particularly important in longitudinal monitoring and comparative studies. For example, in breast IRT, even small differences in patient posture like slight differences in arm abduction may modify breast contour exposure and local skin tension, resulting in measurable changes in regional thermal patterns independent of the underlying pathology [23,24]. In wound and burn assessment, strict standardization of acclimatization conditions and image acquisition timing relative to dressing removal is particularly important, as exposure of the wound surface to ambient air may alter local thermal patterns and thereby affect the thermographic assessment of burn depth and wound inflammation [25]. In brown adipose tissue (BAT) infrared thermography, standardization of image acquisition is particularly important for accurate analysis of the supraclavicular region, where BAT depots are typically located. Small variations in head position, neck exposure, or camera angle may alter visualization of vascular structures and regional heat patterns, potentially leading to overestimation or underestimation of BAT-related thermogenesis [26].

Defining the region of interest (ROI) is critical, as it directly affects the reliability and reproducibility of surface temperature measurements. The choice of ROI determines which pixels of the thermal image are included in the analysis, thereby significantly influencing calculated mean, maximum, or gradient temperature values used for diagnostic purposes. This issue is particularly evident in medical IRT within fields such as breast cancer screening, musculoskeletal disorder assessment, and diabetic foot evaluation, where different ROI delineation approaches (e.g., manual, semi-automatic, or algorithm-based segmentation) may yield divergent results and potentially lead to inconsistent clinical interpretations [27]. Consequently, the absence of harmonized protocols for ROI definition remains one of the major sources of methodological variability in quantitative medical IRT. The growing use of AI-assisted thermographic analysis additionally introduces variability related to segmentation algorithms, training datasets, and preprocessing pipelines.

**Figure 1:**
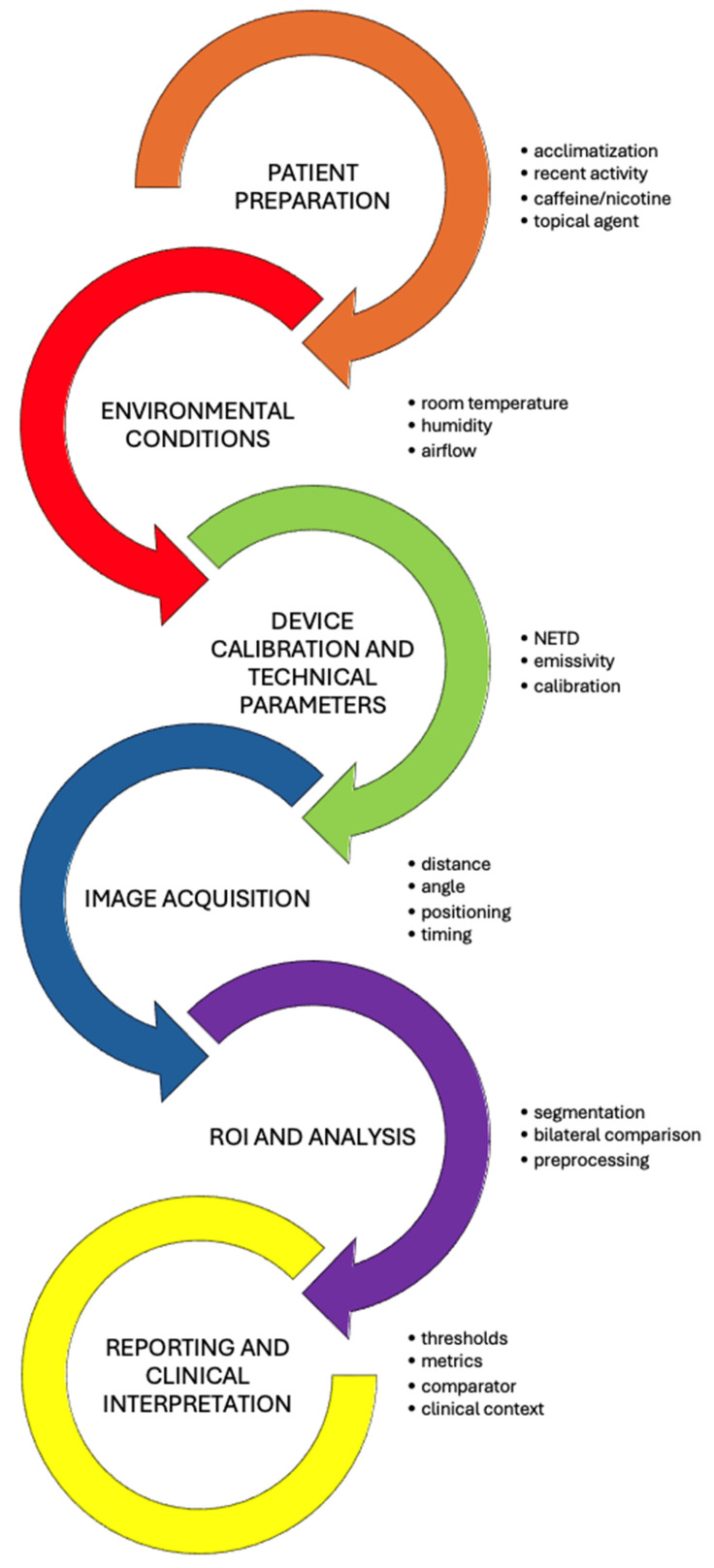
Key stages of the infrared thermography (IRT) workflow and major sources of variability affecting measurement reliability and clinical interpretation.

A thermogram does not represent a direct structural image, but rather an indirect visualization of underlying physiological processes that are highly sensitive to acquisition conditions and analytical methodology. In practice, the lack of unified standards leads to situations where similar temperature distributions may be interpreted differently depending on applied thresholds, data normalization strategies, or selected statistical descriptors. For example, in diabetic foot assessment, a small temperature difference between corresponding regions of both feet may be considered a clinically significant indicator of inflammation in one protocol, while in another it may fall within physiological variability limits, directly influencing decisions regarding preventive interventions for ulcer development [28]. In this context, standardization of reporting—encompassing acquisition conditions as well as the manner of presenting results (e.g., temperature metrics, region definitions, and comparison strategies)—is a prerequisite for transforming IRT from an auxiliary imaging modality into a clinically validated diagnostic tool. This limitation has important clinical implications in diabetic foot monitoring, where preventive interventions are frequently triggered by predefined inter-limb temperature thresholds. For example, some protocols consider a temperature asymmetry exceeding 2.2 °C between corresponding plantar regions as an early marker of inflammatory changes preceding ulceration, whereas other approaches apply different thresholds, averaging methods, or region-of-interest definitions. Consequently, the same thermographic finding may either prompt immediate off-loading and intensified surveillance or be interpreted as physiologic variation, potentially affecting ulcer prevention outcomes [29,30]. Table 1 outlines selected clinical applications of infrared thermography. It places particular emphasis on its added value relative to standard methods, as well as the key sources of false-positive (FP) and false-negative (FN) results and the still-variable maturity of evidence. Figure 1 illustrates the key stages of IRT in medical practice using a basic flowchart.

Over the past decades, several efforts have been undertaken to introduce greater standardization into medical IRT. The International Academy of Clinical Thermology has developed general guidelines concerning infrared imaging, including calibration procedures, environmental control, and measurement reliability. In parallel, discipline-specific recommendations have been proposed by clinical and research groups, particularly in areas such as breast infrared thermography, vascular assessment, and musculoskeletal diagnostics. Despite these advances, full harmonization has not yet been achieved. One of the main challenges is the substantial heterogeneity of clinical applications, which require different acquisition protocols and analytical approaches. Variability in camera technologies, sensitivity, and calibration standards further complicates direct comparison between studies. As a result, while important standardization efforts exist, their adoption remains partial and inconsistent across the field. Table 2 below provides an overview of current standardization proposals in medical IRT, summarizing their scope, key elements, and main limitations.

## 5. Screening, Triage, and Public Health Applications

Infrared thermography has been widely explored in public health contexts as a rapid, contactless tool for large scale temperature screening. Its main advantage is the ability to measure skin temperature remotely, without physical contact, allowing high volume processing triage in settings such as airports, hospitals, workplaces, and border crossings. During outbreaks of infectious diseases, thermal screening systems were deployed globally to identify individuals with elevated body temperature suggestive of fever [33]. However, the limitations of IRT for fever detection have become increasingly evident [34]. Reported sensitivity for detecting fever has varied widely across studies, ranging from approximately 60% to 90%, while specificity also showed substantial variability depending on the device, setting, and threshold used. Critically, asymptomatic and pre-symptomatic individuals—common in SARS-CoV-2 infection—cannot be identified through temperature screening, substantially limiting its epidemiological impact in uncontrolled environments. Moreover, many large screening programs at airports and public venues lacked standardization, calibration procedures, and validated threshold definitions. This resulted in both false positives (e.g., due to environmental heat exposure) and false negatives (e.g., due to antipyretic use or early-stage infection), reducing overall effectiveness [35]. Comparisons with other temperature measurement methods (such as tympanic or oral thermometers) indicate that IRT is generally less accurate.

## 6. Role of Artificial Intelligence and Advanced Analytics in Infrared Thermography

The integration of artificial intelligence (AI) and advanced analytics may open new avenues for the clinical and research applications of IRT. Conventional IRT analysis has traditionally relied on absolute or regional skin temperature measurements and manual interpretation of thermal asymmetries. Advanced analytical methods, especially based on machine learning classification models, have demonstrated promising performance in identifying clinically relevant thermal patterns from IRT images. Supervised and unsupervised machine learning methods have been applied to classify disease states from IRT images across multiple clinical domains.

In diabetic foot assessment, these models may detect localized thermal asymmetries and potentially support early prediction of ulcer development. Based on research findings, AI models can achieve high diagnostic accuracy in the classification of thermal images of diabetic feet with reported classification accuracies frequently exceeding 85–90% in experimental settings. However, most evidence originates from single-center studies with predominantly internal validation [36]. One recent study demonstrated that combining IRT with machine learning techniques enabled differentiation between diabetic patients and a control group with an accuracy of approximately 90%. PCA (Principal Component Analysis) was used to reduce the dimensionality of thermal image data by extracting the most relevant temperature-related features, while SVM (Support Vector Machine) served as a classification algorithm distinguishing diabetic patients from healthy controls based on thermographic patterns [37]. Recent studies indicate the potential of infrared thermography as a screening tool. Explainability approaches, including Grad-CAM-based saliency maps and attention visualisation techniques, may improve transparency of thermographic AI systems and facilitate clinical interpretation of model outputs, although their clinical utility and reliability require further validation [38].

In the case of breast cancer, the application of artificial intelligence combined with infrared thermography is mainly based on the analysis of thermal images using deep learning methods, particularly Convolutional Neural Networks (CNNs) [39]. Recent investigations suggest that machine learning algorithms may support the early detection of breast cancer through the analysis of thermograms with high diagnostic effectiveness, especially as a complementary method to conventional imaging techniques such as mammography, although external validation remains limited [40]. These findings suggest that AI may enhance the diagnostic value of IRT as a non-invasive and contactless screening tool [41].

In musculoskeletal conditions, artificial intelligence is used to analyze thermographic images for the detection of inflammation, muscle overload, joint dysfunction, and rehabilitation monitoring. Evidence suggests that the combination of IRT and AI may support the diagnosis of rheumatic diseases, sports injuries, and muscle overload at a potentially early stage, even before visible structural abnormalities appear [42].

Despite its promising applications, several challenges remain in AI-driven IRT analysis. A key concern is model overconfidence, as algorithms may generate high-confidence predictions even from noisy, incomplete, or biased data. Poor calibration may lead models to assign excessive confidence to uncertain predictions, potentially limiting clinical reliability. Data heterogeneity further limits generalizability, with variations in skin properties, ambient conditions, and device specifications affecting performance [43]. In addition, the usefulness of AI in infrared thermography is strongly dependent on standardization of image acquisition protocols, preprocessing pipelines, region-of-interest (ROI) definition, and annotation procedures. Robust validation through prospective, multicenter studies is therefore essential to establish reproducibility, clinically meaningful thresholds, and real-world performance. A major current limitation is that many AI models in IRT are trained on relatively homogeneous datasets and have not yet undergone robust external validation across centers, devices, and real-world acquisition conditions [44]. Consequently, current AI-based infrared thermography systems shoud be regarded primarily as clinical decision-support tools rather than autonomic diagnostic system. Future research should focus on multimodal data integration, systematic prospective data capture, and transparent model design to maximize clinical impact while minimizing associated risks, as illustrated by the key steps of a typical AI pipeline for infrared thermography summarized in Box 1.

Box 1 **Standard AI pipeline for infrared thermography (IRT)**

A typical artificial intelligence (AI) workflow applied to infrared thermography in medical settings includes the following steps:

1. Data acquisition—standardized thermal image capture under controlled environmental conditions (e.g., ambient temperature, acclimatization time, camera calibration).
2. Pre-processing—image normalization, noise reduction, correction for environmental and device-related variability.
3. Region of interest (ROI) definition—manual, semi-automated, or automated segmentation of anatomically relevant areas.
4. Feature extraction—derivation of quantitative descriptors (e.g., temperature statistics, asymmetry indices, texture features) or use of deep learning for au-tomated feature learning.
5. Model development—training of machine learning or deep learning models for classification, detection, or prediction tasks.
6. Validation and testing—internal validation (e.g., cross-validation) and, ideally, external validation on independent datasets.
7. Clinical interpretation and integration—translation of model outputs into clinically meaningful information and potential integration into decision support systems.

Each step may introduce variability and potential bias, underscoring the need for standardization and robust validation.

## 7. Future Directions: From Snapshot Imaging to Continuous Thermal Intelligence

Infrared thermography may evolve from an episodic imaging modality into a continuous, home-based and clinically integrated monitoring tool within telemedicine and smart healthcare systems. In this paradigm, it would function as a longitudinal sensor of vascular, inflammatory, and metabolic changes rather than a single-time diagnostic test. Central to this concept is the development of individualized thermal baselines, enabling detection of patient-specific deviations that may precede clinical symptoms. When combined with AI, such deviation-based models could support early identification of inflammation, infection, perfusion disorders, or autonomic dysfunction, shifting IRT toward personalized pattern recognition rather than fixed diagnostic thresholds [45].

**Figure 2:**
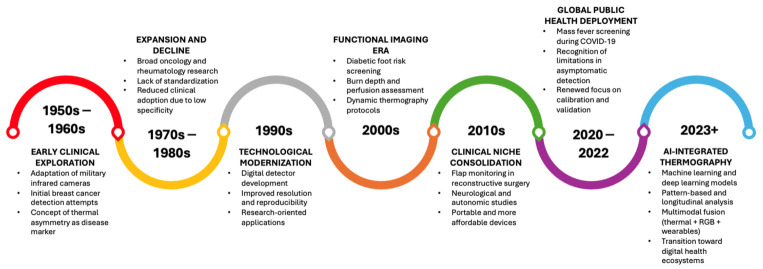
Timeline of Infrared thermography in Medicine.

Integration with telemedicine and hospital systems could enable continuous data acquisition from portable or smartphone-based devices, with automated cloud-based analysis and clinical feedback. Infrared thermography could also complement existing monitoring by providing contactless assessment of microcirculation, wound healing, or post-surgical inflammation, and when combined with electronic health records and wearable support a multimodal view of patient status [46]. However, translation into clinical practice requires rigorous prospective validation across diverse populations and real-world conditions. Models trained on homogeneous datasets often show reduced generalizability when exposed to variability in skin properties, devices, and acquisition protocols. In addition, risks such as algorithmic overconfidence and automation bias must be addressed to ensure AI remains a decision-support tool rather than a replacement for clinical judgment. Ethical and governance issues—including data privacy, transparency, and equitable access—are also essential for safe and sustainable implementation [47]. Figure 2 illustrates the development of IRT in medicine over the past 70 years.

## 8. Conclusions

IRT provides noninvasive real-time assessment as a physiological sensor for long-term monitoring. Its principal strengths are contactless acquisition, functional insight, and sensitivity to physiological changes that may precede structural alterations. When applied using standardized protocols and interpreted alongside clinical findings, IRT can serve as a valuable supportive tool. However, it cannot yet function as a standalone diagnostic modality in most clinical domains, and current evidence does not support replacing established imaging or screening standards. Performance remains limited by environmental variability, inter-individual differences, lack of universally accepted thresholds, and insufficient large scale prospective validation. To advance the field, research investment should focus on multicenter validation studies and the worldwide alignment of existing thermography protocols. Equally important is the development of AI-driven, pattern-based and longitudinal analytical models grounded in personalized baselines. Even small deviations in factors like patient preparation, environmental conditions, camera calibration, and image acquisition or analysis procedures may affect thermal patterns and reduce reproducibility, highlighting the need for more standardized and globally harmonized protocols. While multiple factors contribute to the limited clinical adoption of IRT, standardization appears to be among the most actionable barriers and therefore a key target for facilitating its translation into routine practice. Integration with multimodal digital health systems may ultimately determine whether IRT evolves from a promising adjunct into a scalable component of precision and preventive medicine.

## Figures and Tables

**Table 1:** Key clinical applications of infrared thermography (IRT): comparison with standard approaches, added value, and limitations.

Clinical Scenario	Standard Comparator	Possible Added Value of IRT	Major FP/FN Risks	Maturity of Evidence	References
Diabetic foot monitoring	Clinical examination, temperature probes, pressure assessment	Early detection of inflammation preceding ulceration; potential for remote and home monitoring	FP: ambient conditions, recent activity; FN: neuropathy, impaired perfusion	Moderate (promising for prevention)	[12,27,28,29,30]
Breast cancer (adjunct use)	Mammography, ultrasound, MRI	Non-contact, radiation-free adjunct; detection of vascular/thermal asymmetry	FP: benign inflammation, hormonal influences; FN: small or deep tumors	Low–moderate (not standalone)	[4,23,24]
Inflammatory joint diseases	Clinical exam, ultrasound, MRI	Rapid, non-invasive assessment of joint inflammation; objective thermal patterns	FP: skin temperature variability; FN: low-grade or deep inflammation	Moderate (supportive role)	[17,30,31,32]
Wound and burn assessment	Clinical evaluation, imaging (selected cases)	Monitoring perfusion and healing; early detection of complications	FP: dressings, environment. FN: deep tissue processes	Moderate	[25]
Evaluation of brown adipose tissue (BAT) activity	PET/CT (FDG uptake)	Non-invasive, radiation-free, real-time assessment of thermal activity	FP: Elevated skin temperature due to ambient conditions, recent physical activity, or increased skin blood flow unrelated to BAT	Emerging/limited clinical validation	[26]

Maturity of evidence reflects the overall quantity, consistency, and clinical validation of published evidence rather than a formal evidence-grading framework. “Emerging” indicates predominantly exploratory studies with limited clinical validation; “Low–moderate” indicates a growing but inconsistent evidence base without established clinical utility; and “Moderate” indicates relatively consistent evidence supporting an adjunctive clinical role, although not sufficient for standalone diagnostic use.

**Table 2:** Overview of current standardization proposals in medical infrared thermography.

Standard/Guideline	Scope (Clinical/Research)	Standardization Coverage (What Is Explicitly Addressed)	Remaining Gaps (What Is Not Sufficiently Regulated)
International Organization for Standardization (Infrared imaging standards)	Research/technical (partially clinical)	Technical performance: camera calibration, measurement accuracy, environmental conditions	Lack of clinical context; no guidance on patient preparation, ROI definition, or clinical interpretation
American Society for Testing and Materials (Infrared thermography guidelines)	Research/technical	Equipment performance, acquisition procedures, quality assurance frameworks	Limited applicability to medical settings; no standardized clinical protocols or interpretation criteria
European Association of Thermology recommendations	Clinical/Research	Patient preparation, acquisition protocols, qualitative interpretation approaches	Limited standardization of quantitative analysis; variability in implementation; no universally accepted thresholds
American Academy of Thermology guidelines	Clinical	Clinical protocols, reporting standards, safety considerations, basic interpretation guidance	Limited international adoption; insufficient validation across different clinical indications
Musculoskeletal thermography protocols	Research/Emerging clinical use	ROI definition, bilateral comparison, functional and provocation testing, dynamic assessment concepts	No consensus on diagnostic thresholds; limited reproducibility; lack of unified protocols for dynamic vs. static assessments

## Data Availability

No new data were created or analyzed in this study.
